# Supplementary figures and images for: The Chilean burden of disability-adjusted life years due to cardiovascular diseases: Results from the Global Burden of Disease Study 2021
Source: PLoS One. 2025 Jun 18;20(6):e0325519. doi: 10.1371/journal.pone.0325519 (PMC12176180; doi:10.1371/journal.pone.0325519)

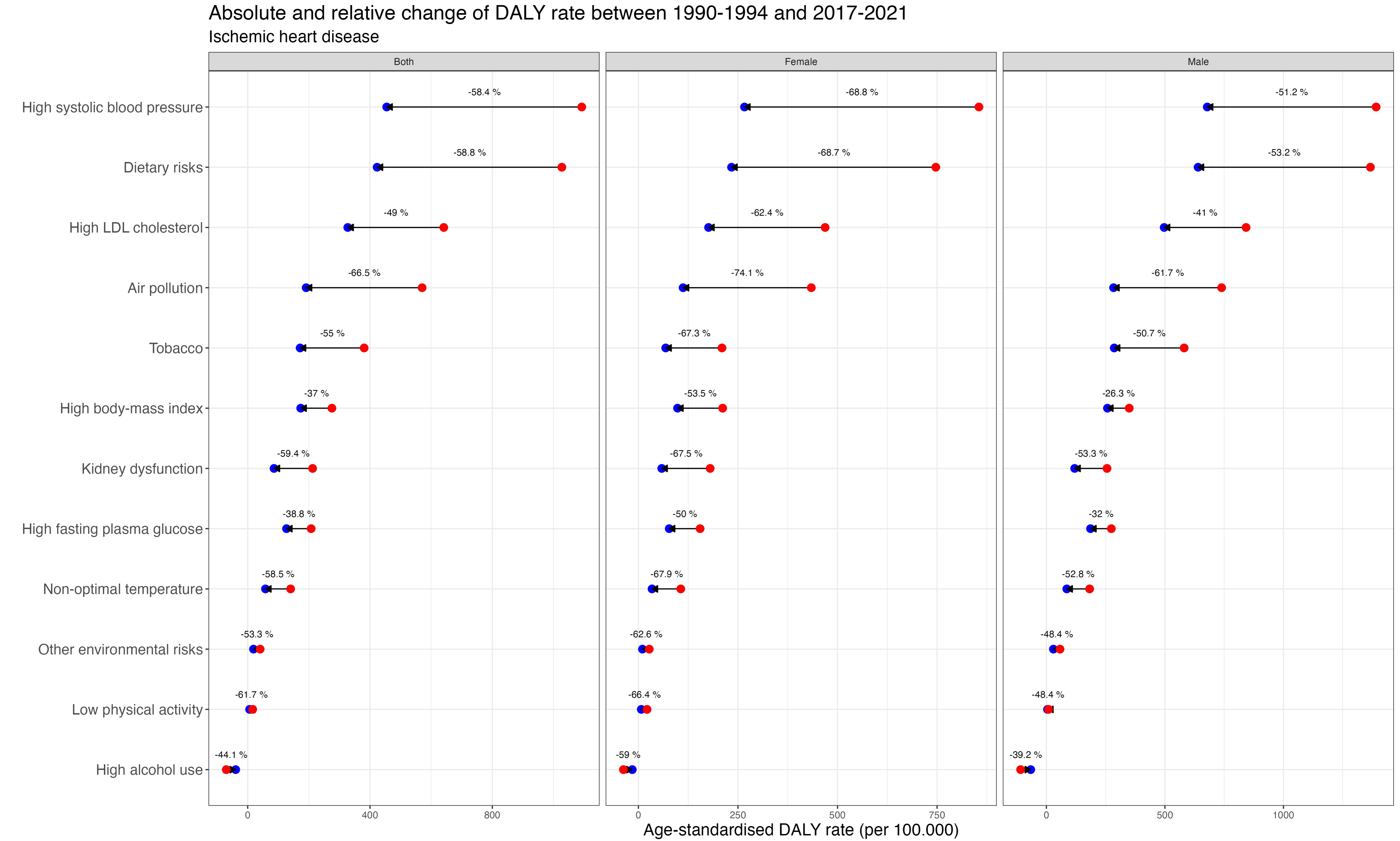

Supplement: S1 Fig — The red dot represents the mean 5-year period for 1990–1994, and the blue dot represents the 2017–2021 period. (TIF) [file pone.0325519.s001.tif]

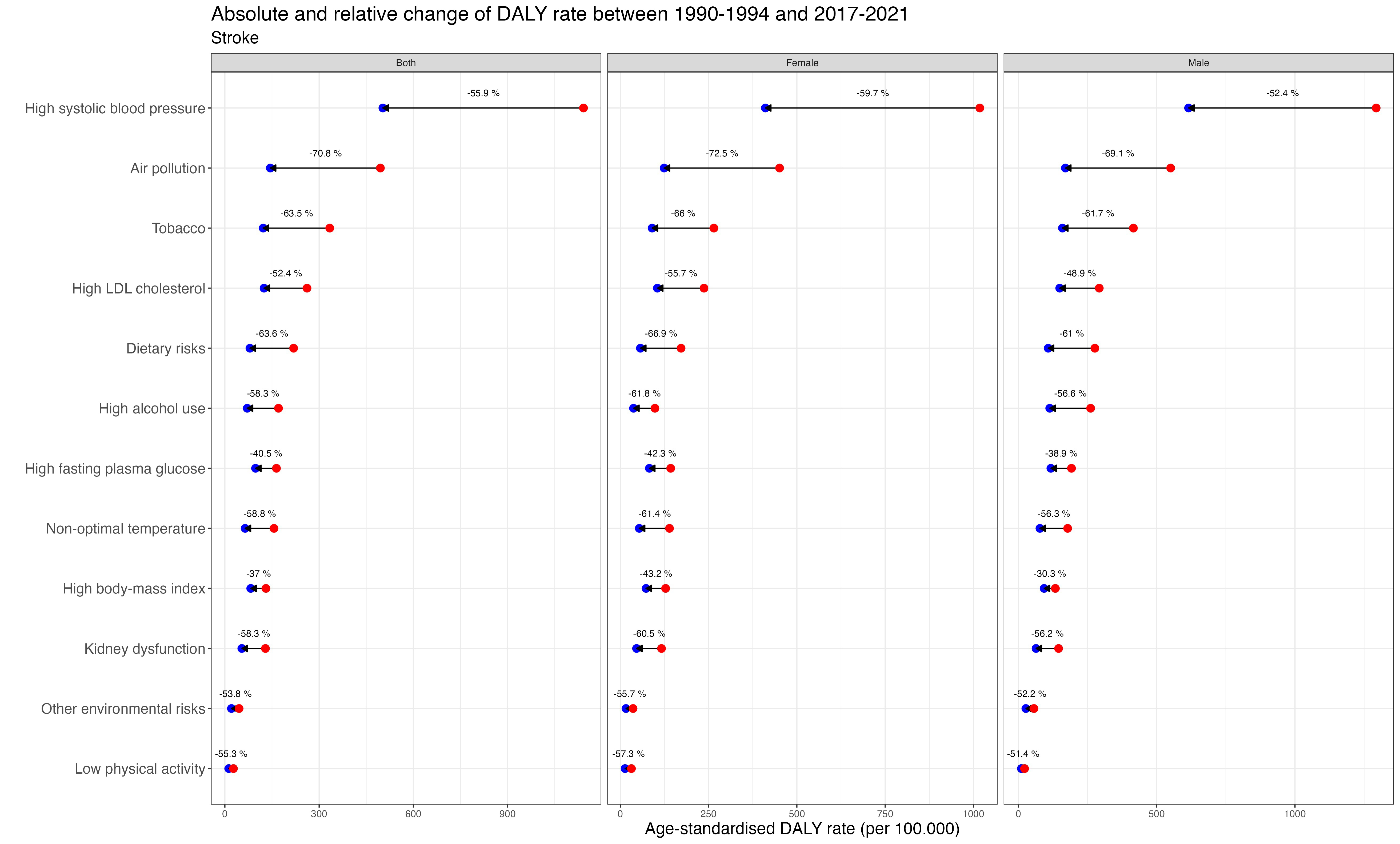

Supplement: S2 Fig — The red dot represents the mean 5-year period for 1990–1994, and the blue dot represents the 2017–2021 period. (TIF) [file pone.0325519.s002.tif]

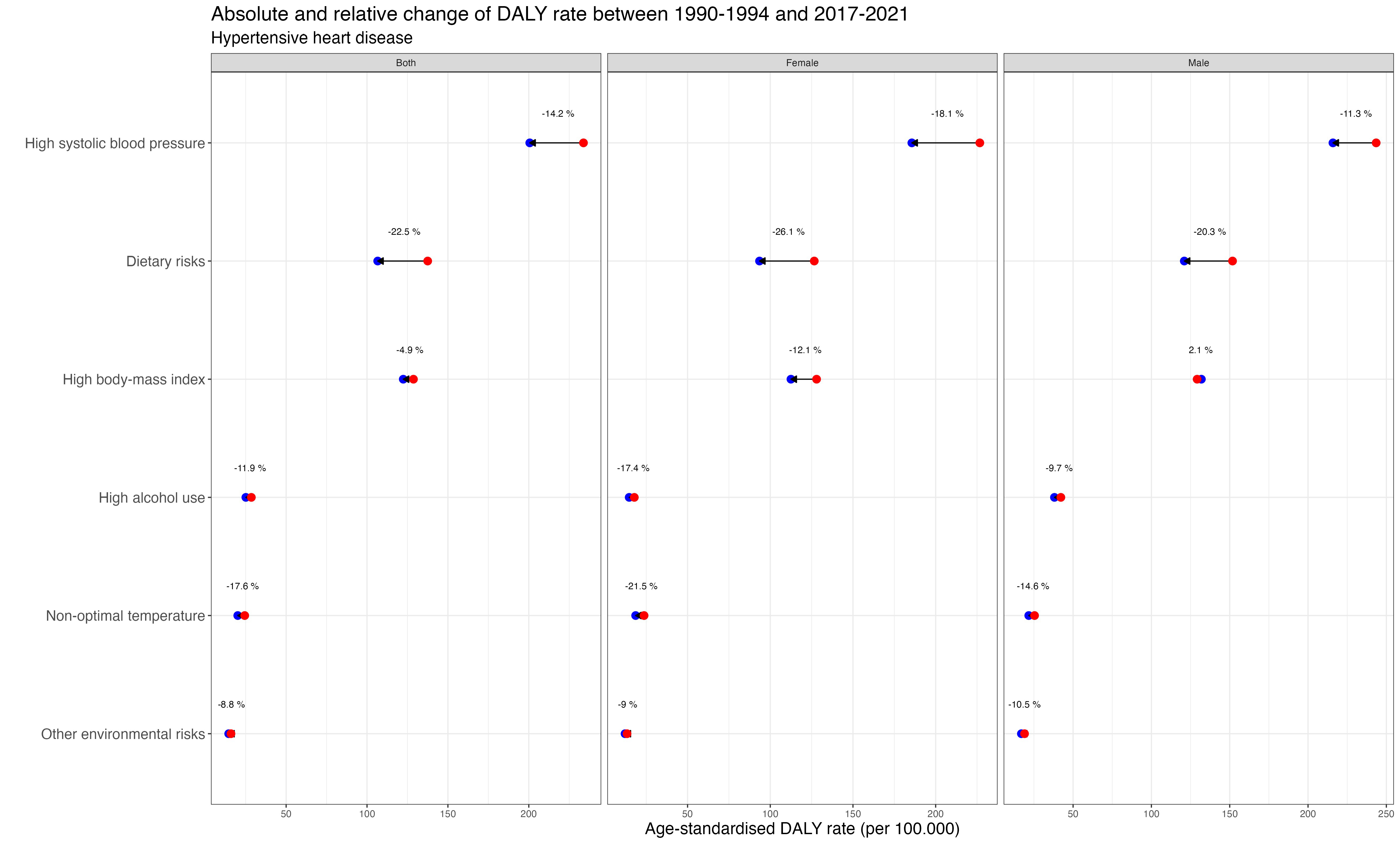

Supplement: S3 Fig — The red dot represents the mean 5-year period for 1990–1994, and the blue dot represents the 2017–2021 period. (TIF) [file pone.0325519.s003.tif]

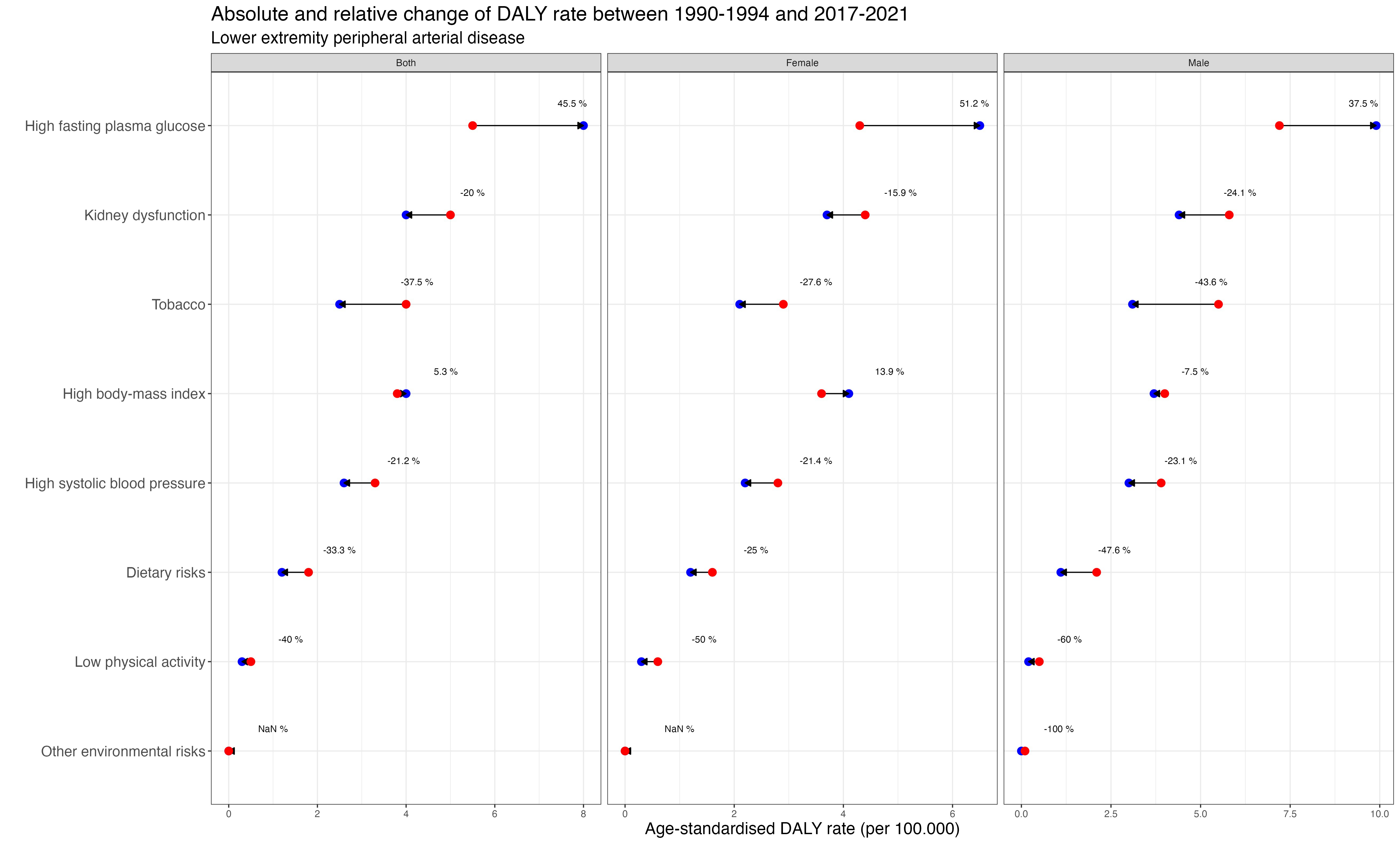

Supplement: S4 Fig — The red dot represents the mean 5-year period for 1990–1994, and the blue dot represents the 2017–2021 period. (TIF) [file pone.0325519.s004.tif]

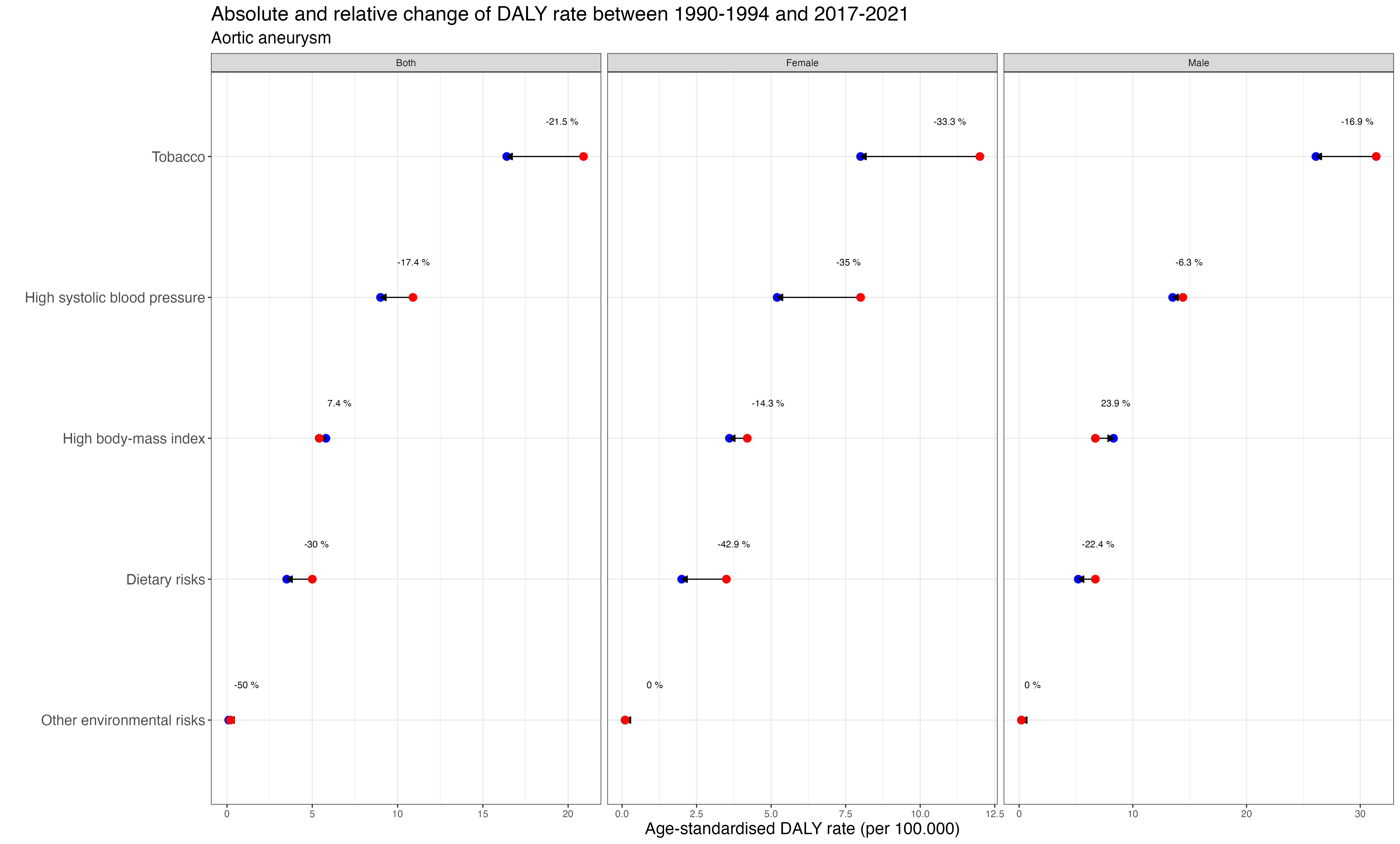

Supplement: S5 Fig — The red dot represents the mean 5-year period for 1990–1994, and the blue dot represents the 2017–2021 period. (TIF) [file pone.0325519.s005.tif]

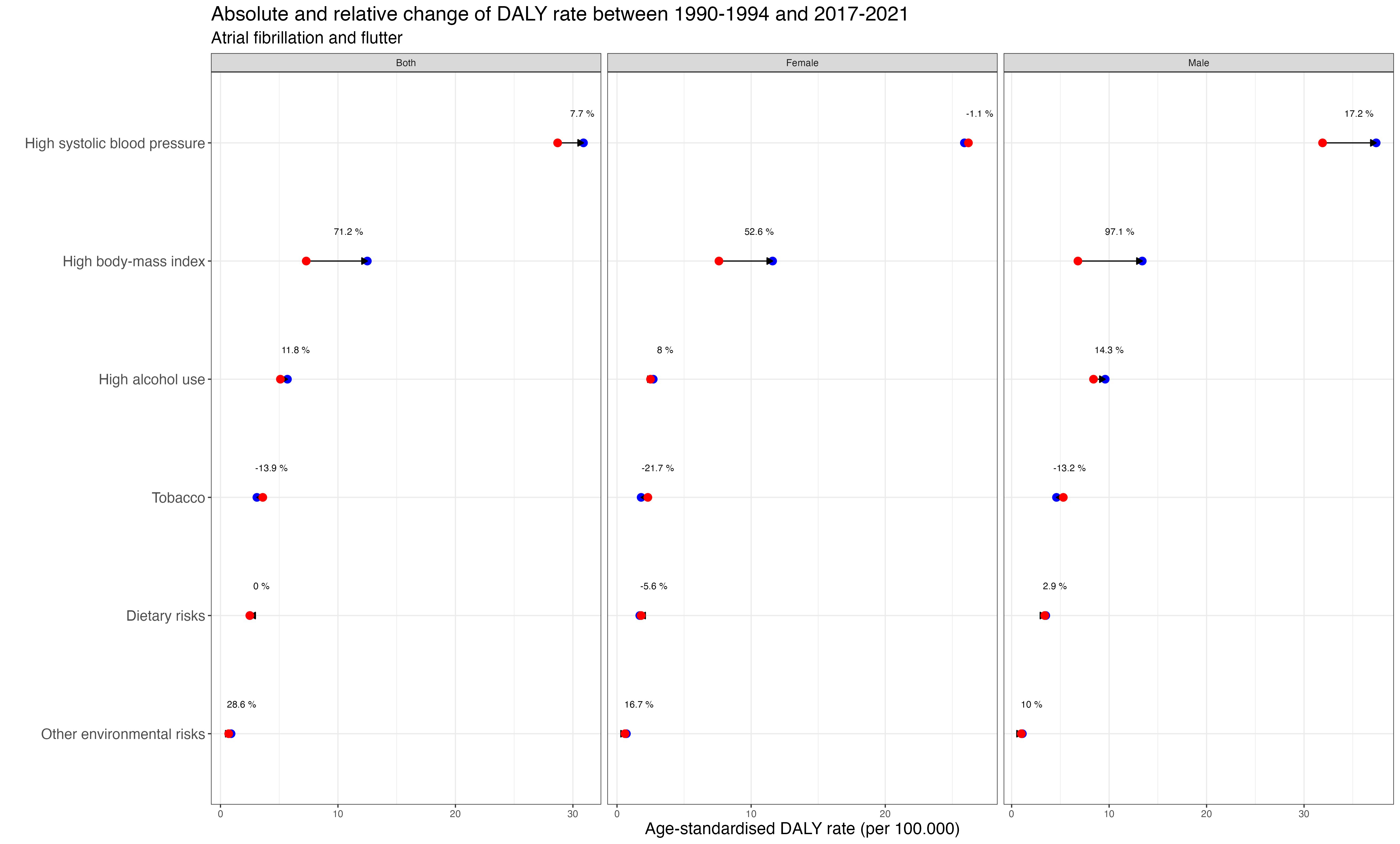

Supplement: S6 Fig — The red dot represents the mean 5-year period for 1990–1994, and the blue dot represents the 2017–2021 period. (TIF) [file pone.0325519.s006.tif]

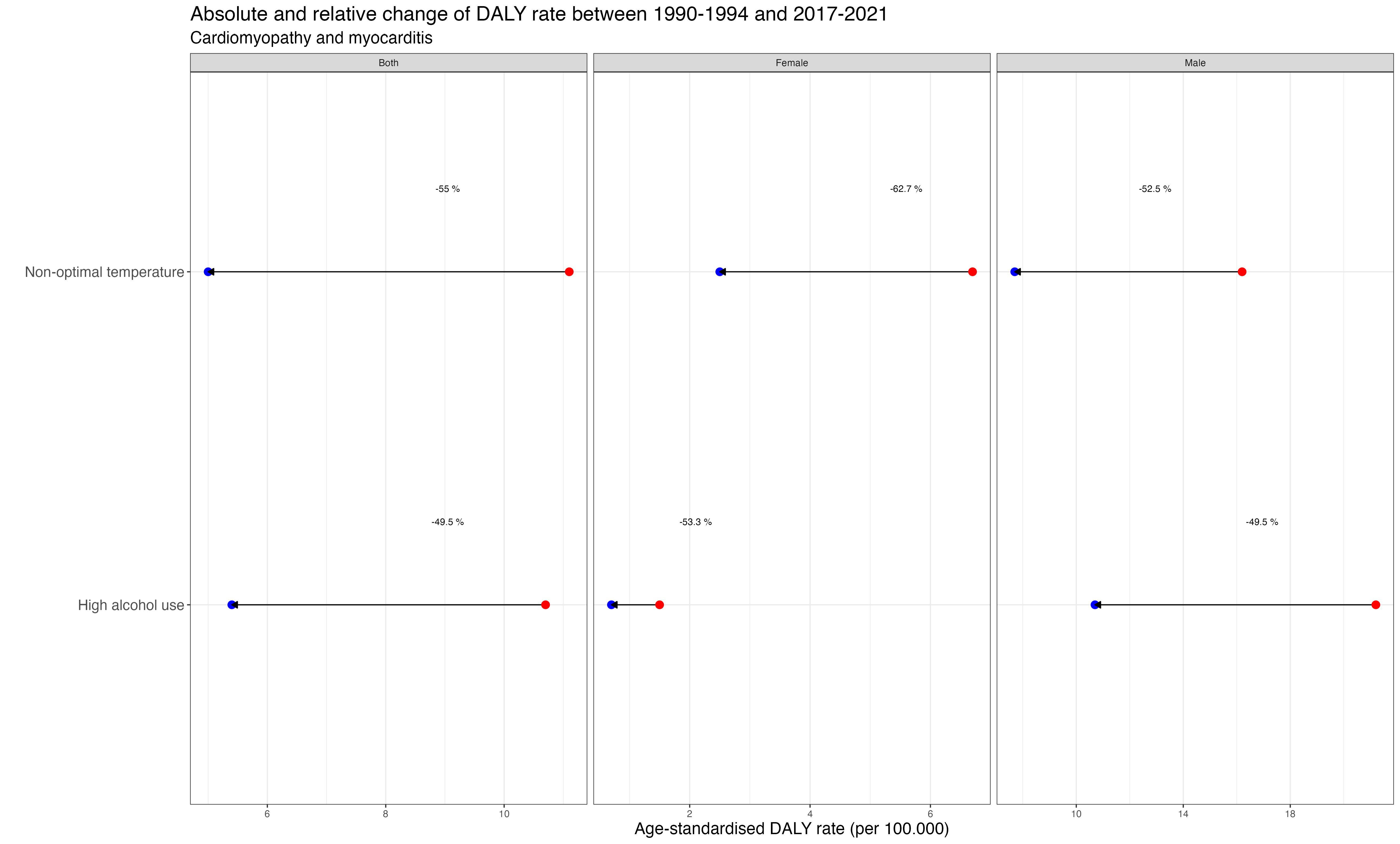

Supplement: S7 Fig — The red dot represents the mean 5-year period for 1990–1994, and the blue dot represents the 2017–2021 period. (TIF) [file pone.0325519.s007.tif]
